# Predictors of influenza severity among hospitalized adults with laboratory confirmed influenza: Analysis of nine influenza seasons from the Valencia region, Spain

**DOI:** 10.1111/irv.12985

**Published:** 2022-04-12

**Authors:** Nieves Derqui, Joshua Nealon, Ainara Mira‐Iglesias, Javier Díez‐Domingo, Cedric Mahé, Sandra S. Chaves

**Affiliations:** ^1^ Sanofi Pasteur Lyon France; ^2^ Univ Lyon Université Claude Bernard Lyon 1 Villeurbanne France; ^3^ School of Public Health, Li Ka Shing Faculty of Medicine The University of Hong Kong Hong Kong China; ^4^ Fundación para el Fomento de la Investigación Sanitaria y Biomédica de la Comunitat Valenciana (FISABIO‐Public Health) Valencia Spain

**Keywords:** comorbidity, death, influenza, severity, Spain

## Abstract

**Purpose:**

Influenza hospitalizations contribute substantially to healthcare disruption. We explored the impact of ageing, comorbidities and other risk factors to better understand associations with severe clinical outcomes in adults hospitalized with influenza.

**Methods:**

We analysed multi‐season data from adults ≥18 years, hospitalized with laboratory‐confirmed influenza in Valencia, Spain. Severity was defined as intensive care unit (ICU) admission, assisted ventilation and/or death. Generalized estimating equations were used to estimate associations between risk factors and severity. Rate of hospital discharge was analysed with a cumulative incidence function.

**Results:**

Only 26% of influenza patients had their primary discharge diagnosis coded as influenza. Comorbidities were associated with severity among adults aged 50–79 years, with the highest odds ratio (OR) in patients with ≥3 comorbidities aged 50–64 years (OR = 6.7; 95% CI: 1.0–44.6). Morbid obesity and functional dependencies were also identified risk factors (ORs varying from 3 to 5 depending on age). The presence of increasing numbers of comorbidities was associated with prolonged hospital stay.

**Conclusions:**

Influenza clinical outcomes are aggravated by the presence of comorbidities and ageing. Increased awareness of influenza among hospitalized patients could prompt clinical and public health interventions to reduce associated burden.

## 1 INTRODUCTION

Seasonal influenza is a viral infection of global public health importance, disproportionately affecting those at extremes of age, smokers, pregnant women and those with pre‐existing medical conditions.^1^, ^2^, ^3^, ^4^ Severe outcomes can include prolonged hospitalization, the need for mechanical ventilation and/or intensive care unit (ICU) admission, longer‐term functional and quality‐of‐life decline, progression to pneumonia or other secondary outcomes and even death.^5^, ^6^, ^7^, ^8^

In Spain, influenza vaccination is recommended for adults aged over 65 or 60 (depending on region). In the Valencia region, those aged over 60 were recommended for vaccination until the 2017/2018 season when this was revised to include only adults aged over 65. Influenza vaccination is also recommended for individuals with a range of chronic medical conditions and for occupational sectors that may predispose to elevated risk of exposure.^9^ In the 2013/2014 and 2014/2015 influenza seasons, vaccine coverage rate among older adults was 54%, and—in common with nearly all European countries—further efforts will be required to reach the global recommendations to achieve vaccine coverage of ≥75% among those ≥65 years.^9^, ^10^ Improved influenza vaccines with better immunogenicity,^11^ efficacy^12^, ^13^ and effectiveness^14^, ^15^ than standard dose vaccines in older age groups are/will soon be available in European countries. These vaccines may also provide better protection for adults with comorbidities contributing to better overall health outcomes.

We analysed influenza hospitalization data from 2010/2011 through 2018/2019 influenza seasons from an integrated surveillance network, operating since 2010 in the Valencia region of Spain.^16^ We identified predictors of severe outcomes associated with influenza in hospitalized adult patients of different age groups and described their clinical presentation and progression to better understand the contribution of comorbidity and ageing to severe influenza presentation.

## 2 METHODS

### 2.1 Ethical statement

The Ethics Research Committee of the Dirección General de Salud Pública‐Centro Superior de Investigación en Salud Pública approved the protocol, and all patients provided written informed consent before their inclusion.

### 2.2 Data source and study protocol

We analysed hospital‐based influenza surveillance data collected from the Valencia region of Spain during influenza seasons 2010/2011 through 2018/2019, collected following a standard prospective active surveillance study protocol as previously described.^16^, ^17^ Briefly, patients presenting with a protocol‐defined respiratory, cardiovascular or other specified complaints, hospitalized for minimum one night, non‐institutionalized and resident in the hospital catchment area were eligible for screening. Those presenting with at least one respiratory and one systemic symptom with an onset of <7 days, as per the European Centre for Disease Prevention and Control influenza‐like‐illness (ILI) case definition, were invited to join the study.^18^ After informed consent, detailed clinical and demographic data were gathered through patient interview and medical record abstraction into a study database. Pharyngeal and nasopharyngeal swab samples from all study participants were collected and tested for influenza by reverse transcription polymerase chain reaction (RT‐PCR). Patients were followed up during hospitalization with collection of data on clinical progression, treatments and discharge.

### 2.3 Definitions and categorization

Our analysis focused on hospitalized adult patients aged 18 years or older that were identified with laboratory‐confirmed influenza virus infection. Study covariates were grouped into commonly applied categories: Age was categorized into four strata (18–49, 50–64, 65–79 and ≥80 years); body mass index (BMI) was grouped into underweight (<18.5), normal (18.5 to <25), overweight (25 to <30), obese (30 to <40) and morbidly obese (≥40)^19^; and functional dependency, in patients aged ≥65 for whom these data were captured, was grouped according to the Barthel Index: total (0–15), severe (20–35), moderate (40–55), mild (60–90) and minimal (95–100).^20^ Information on influenza vaccination status was ascertained from public health vaccination records, considering individuals as ‘vaccinated’ if they had received influenza vaccine in the season of recruitment. We considered a patient was treated with antiviral therapy when treatment was initiated either before or during hospitalization.

To analyse the impact of comorbidities (listed in Table 1), patients were categorized into (i) those with no underlying chronic medical conditions and (ii) those with one, (iii) two and (iv) three or more conditions. Selected comorbidities were also evaluated separately as risk factors by comparing patients with each comorbidity to those without.

**TABLE 1:** Demographic and clinical characteristics of hospitalized adults with laboratory‐confirmed influenza, Valencia, 2010–2019

Characteristics	Patients, no. (%)			*P*‐value
	Total (*n* = 3180)	Not severe (*n* = 2910)	Severe (*n* = 270)	
Number of comorbidities				0.002
None	493 (16)	460 (16)	33 (12)	
1	961 (30)	895 (31)	66 (24)	
2	828 (26)	759 (26)	69 (26)	
≥3	898 (28)	796 (27)	102 (38)	
Sex				0.300
Male	1674 (53)	1540 (53)	134 (50)	
Female	1506 (47)	1370 (47)	136 (50)	
Age group				*P* < 0.001
18–49	303 (10)	287 (10)	16 (6)	
50–64	486 (15)	451 (15)	35 (13)	
65–79	1191 (37)	1107 (38)	84 (31)	
≥80	1200 (38)	1065 (37)	135 (50)	
Smoking				0.704
Current	541 (17)	500 (17)	41 (15)	
Former	1078 (34)	984 (34)	94 (35)	
Never	1561 (49)	1426 (49)	135 (50)	
BMI				0.126
Underweight	64 (2)	56 (2)	8 (3)	
Normal	944 (30)	853 (29)	91 (34)	
Overweight	1254 (39)	1162 (40)	92 (34)	
Obese	822 (26)	755 (26)	67 (25)	
Morbid obese	96 (3)	84 (3)	12 (4)	
Functional dependency^a^				*P* < 0.001
Total	154 (6)	118 (5)	36 (16)	
Severe	67 (3)	59 (3)	8 (4)	
Moderate	145 (6)	129 (6)	16 (7)	
Mild	474 (20)	427 (20)	47 (21)	
Minimal	1559 (65)	1447 (66)	112 (51)	
Cardiovascular disease				0.011
Yes	1474 (46)	1329 (46)	145 (54)	
No	1706 (54)	1581 (54)	125 (46)	
Asthma				0.074
Yes	324 (10)	305 (10)	19 (7)	
No	2856 (90)	2605 (90)	251 (93)	
Other chronic respiratory disease				0.144
Yes	1039 (33)	940 (32)	99 (37)	
No	2141 (67)	1970 (68)	171 (63)	
Chronic endocrine system disease				0.185
Yes	1154 (36)	1046 (36)	108 (40)	
No	2026 (64)	1864 (64)	162 (60)	
Anaemia				0.326
Yes	322 (10)	290 (10)	32 (12)	
No	2858 (90)	2620 (90)	238 (88)	
Chronic liver disease				0.585
Yes	111 (3)	100 (3)	11 (4)	
No	3069 (97)	2810 (97)	259 (96)	
Chronic renal disease				*P* < 0.001
Yes	489 (15)	426 (15)	63 (23)	
No	2691 (85)	2484 (85)	207 (77)	
Immunopathology				0.371
Yes	153 (5)	137 (5)	16 (6)	
No	3027 (95)	2773 (95)	254 (94)	
Neurological disorders				0.281
Yes	263 (8)	236 (8)	27 (10)	
No	2917 (92)	2674 (92)	243 (90)	
Neoplasia				0.568
Yes	170 (7)	153 (7)	17 (8)	
No	2352 (93)	2147 (93)	205 (92)	
Vaccination status				0.806
Yes	1803 (57)	1648 (57)	155 (57)	
No	1377 (43)	1262 (43)	115 (43)	
Antiviral use				0.679
Yes	499 (16)	459 (16)	40 (15)	
No	2681 (84)	2451 (84)	230 (85)	
Virus strain				0.330
H1N1pdm09	747 (23)	672 (23)	75 (28)	
H3N2	1840 (58)	1692 (58)	148 (55)	
B	464 (15)	429 (15)	35 (13)	
Untyped	129 (4)	117 (4)	12 (4)	
Season				*P* < 0.001
2010/2011	98 (3)	90 (3)	8 (3)	
2011/2012	560 (18)	520 (18)	40 (15)	
2012/2013	187 (6)	180 (6)	7 (3)	
2013/2014	303 (10)	280 (10)	23 (9)	
2014/2015	658 (21)	620 (21)	38 (14)	
2015/2016	245 (8)	227 (8)	18 (7)	
2016/2017	231 (7)	211 (7)	20 (7)	
2017/2018	573 (18)	497 (17)	76 (28)	
2018/2019	325 (10)	285 (10)	40 (15)	

Abbreviation: BMI, body mass index.

^a^ Barthel Index data only available for subjects ≥65 years. BMI categories were defined by underweight (<18.5), normal (18.5 to <25), overweight (25 to <30), obese (30 to <40) and morbid obese (≥40). *P*‐values derived from Pearson's chi‐squared test.

Primary discharge diagnoses associated with each hospitalization were described based on International Classification of Diseases (ICD) codes. Because discharge diagnoses were recorded in ICD‐9 and ICD‐10 codes depending on the season, diagnoses in ICD‐9 were converted to corresponding ICD‐10 diagnostic groups for analysis (Table S1).

We defined severe clinical outcomes using a binary composite indicator based on feedback from site investigators and review of the literature. The indicator included either ICU admission,^3^, ^5^, ^21^ mechanical ventilation or extracorporeal membrane oxygenation (ECMO)^21^, ^22^, ^23^ or death at any time during the patient's hospitalization.^21^, ^22^

### 2.4 Severity risk factor analysis

Univariate associations between potential risk factors and influenza severe outcome, as defined by the indicator above, for the entire study population were assessed using Pearson's chi‐squared test. Adjusted multivariable associations were estimated using generalized estimating equations (GEE).^24^ Our model assumed both a correlation within hospitals (different clinical practices) and within seasons (different strain circulation and severity).^24^ To analyse the contribution of comorbidities independently from age, we developed parsimonious models for each age group to estimate adjusted odds ratios (OR) for severity and 95% confidence intervals (CI) using a robust covariance estimator. Model selection was performed, explicitly retaining information on the number of comorbidities as the primary research question, by removing variables if (i) they were not confounding the relationship between severity and comorbidity (i.e. did not change the effect size by >10%); (ii) they were identified as colinear with other exposure variables (evidenced by increasing standard errors); or (iii) they did not meet the prespecified level for retention (*P*‐value of <0.2). Goodness of fit of final versus full models was evaluated by a quasi‐likelihood based variation of the Akaike Information Criterion using the method proposed by Pan.^25^

### 2.5 Length of stay analysis

We also explored length of hospital stay as a proxy for severity. Association between median length of hospitalization with increasing age (age groups) was compared with the non‐parametric test for trend across ordered groups. A cumulative incidence function was developed to estimate the hazard ratio of hospital discharge according to (i) age group and (ii) comorbid status, considering death as a competing risk, after adjustment for variables retained in final GEE models for each age group, using the method of Fine and Gray.^26^

### 2.6 Software

Statistical analyses were performed with Stata 15.0 (StataCorp LLC, College Station, Texas).

## 3 RESULTS

### 3.1 Description of the population

During the influenza seasons 2010/2011–2018/2019, there were 22 980 hospitalized patients enrolled in the yearly surveillance study. Of those, 3180 were adults with laboratory‐confirmed influenza that were further included in this analysis. Baseline characteristics of study population overall and by severity are described in Table 1. Most (75%) patients were aged ≥65 years, and 84% had ≥1 comorbidity. This proportion varied by age: In the 18–49 year group, 48% had ≥1 comorbidity, rising to 79%, 88% and 90% in 50–64, 65–79 and ≥80 years, respectively (Table S2). Cardiovascular diseases were the most common comorbidity (1474 subjects; 46%), followed by chronic endocrine system diseases (1154; 36%) and chronic respiratory diseases (1039; 33%). The use of influenza antiviral therapy was 16% and did not change based on severity.

Overall, 270 (8.5%) patients experienced ≥1 severe clinical outcome that included 75 ICU admissions, 87 mechanical ventilation, 7 ECMO and 149 deaths. Among those who died during hospitalization, 68% were aged ≥80 years (Table 2). Increasing percentages of severity were observed with increasing age: from 6% among the youngest age group to 50% in patients ≥80 years. Severity also increased with increased number of comorbidities; 38% of patients with severe outcomes had ≥3 comorbidities compared with 27% of those non‐severe (Table 1). For patients aged ≥65 years, where data on Barthel Index were available, total dependency accounted for 16% of severe cases compared with 5% among the non‐severe population (*P* < 0.001). Patients with cardiovascular and chronic renal diseases experienced a significantly higher frequency of severe outcomes compared with those without these conditions. Age‐stratified frequencies of patient characteristics are available in Table S2.

**TABLE 2:** Description of outcomes following hospital admission among hospitalized adults with laboratory‐confirmed influenza, Valencia, 2010–2019

	Patients, no. (%)					Length of hospitalization (days)	
	Total severe (*n* = 270)	ICU admissions (*n* = 75)	Mechanical ventilation (*n* = 87)	ECMO (*n* = 7)	In‐hospital death (*n* = 149)	Median	IQR
Age group							
18–49	16 (6)	14 (19)	5 (6)	2 (29)	1 (1)	5	4–8
50–64	35 (13)	23 (31)	14 (16)	2 (29)	8 (5)	7	5–10
65–79	84 (31)	24 (32)	35 (40)	1 (14)	39 (26)	7	5–9
≥80	135 (50)	14 (19)	33 (38)	2 (29)	101 (68)	7	5–10

Abbreviations: ECMO, extracorporeal membrane oxygenation; ICU, intensive care unit.

### 3.2 Primary discharge diagnoses of hospitalized patients

Information on primary diagnoses was available for 3087 subjects (>97% of the study population), described in Table 3. Most were of respiratory cause (86%), of which influenza was the most common (920 subjects, 30%). However, only 26% of the laboratory confirmed influenza patients had their primary discharge diagnosis coded as influenza. Pneumonia was recorded for 470 subjects (15%), whereas 456 (15%) and 586 patients (19%) were recorded with chronic respiratory and other respiratory diseases, respectively. Non‐respiratory diagnoses were less frequent: 142 patients (5%) had recorded circulatory events including heart attack and cardiac insufficiency (Tables 3 and S1). No substantial differences in discharge diagnosis frequency were observed by age group/severity.

**TABLE 3:** Description of discharge diagnoses of hospitalized adults with laboratory‐confirmed influenza, Valencia, 2010–2019

Discharge diagnoses	Total (*n* = 3087)	Patients, no. (%)					
		Age group distribution				Severe distribution	
		18–49 years (*n* = 297)	50–64 years (*n* = 472)	65–79 years (*n* = 1154)	≥80 years (*n* = 1164)	Not severe (*n* = 2826)	Severe (*n* = 261)
Respiratory outcomes							
Acute upper respiratory infections	195 (6)	15 (5)	23 (5)	70 (6)	87 (7)	187 (7)	8 (3)
Influenza	920 (30)	104 (35)	152 (32)	334 (29)	330 (28)	824 (29)	96 (37)
Pneumonia	470 (15)	59 (20)	60 (13)	177 (15)	174 (15)	428 (15)	42 (16)
Other upper and lower respiratory tract diseases	40 (1)	1 (0)	3 (1)	10 (1)	26 (2)	36 (1)	4 (2)
Chronic respiratory diseases	456 (15)	42 (14)	89 (19)	204 (18)	121 (10)	430 (15)	26 (10)
Other respiratory diseases	586 (19)	35 (12)	90 (19)	119 (17)	262 (23)	536 (19)	50 (19)
Non‐respiratory outcomes							
Other infectious diseases	27 (1)	6 (2)	2 (0)	5 (0)	14 (1)	23 (1)	4 (2)
Neoplasms	11 (0)	0 (0)	1 (0)	6 (1)	4 (0)	9 (0)	2 (1)
Endocrine system diseases	20 (1)	5 (2)	3 (1)	6 (1)	6 (1)	16 (1)	4 (2)
Circulatory system diseases	142 (5)	4 (1)	18 (4)	55 (5)	65 (6)	127 (4)	15 (6)
Mental disorders	7 (0)	1 (0)	0 (0)	2 (0)	4 (0)	7 (0)	0 (0)
Nervous system diseases	8 (0)	1 (0)	1 (0)	2 (0)	4 (0)	8 (0)	0 (0)
Digestive system diseases	3 (0)	0 (0)	2 (0)	0 (0)	1 (0)	3 (0)	0 (0)
Genitourinary system diseases	18 (1)	1 (0)	2 (0)	11 (1)	4 (0)	18 (1)	0 (0)
Skin tissue diseases	0 (0)	0 (0)	0 (0)	0 (0)	0 (0)	0 (0)	0 (0)
Musculoskeletal and connective tissue diseases	1 (0)	0 (0)	1 (0)	0 (0)	0 (0)	1 (0)	0 (0)
Pregnancy, congenital malformations and other related	4 (0)	4 (1)	0 (0)	0 (0)	0 (0)	4 (0)	0 (0)
Various	179 (6)	19 (6)	25 (5)	73 (6)	62 (5)	169 (6)	10 (4)

### 3.3 Multivariate risk factor analysis

Model selection strategy was based on four GEE models (one per age group). In addition to number of comorbidities that was explicitly retained, backward selection retained different combinations of sex, smoking status, BMI, functional dependency, influenza vaccination status and infecting virus lineage as variables in final models, all of which demonstrated better model fit and parsimony through the QIC test than full models (data not shown).

**FIGURE 1:**
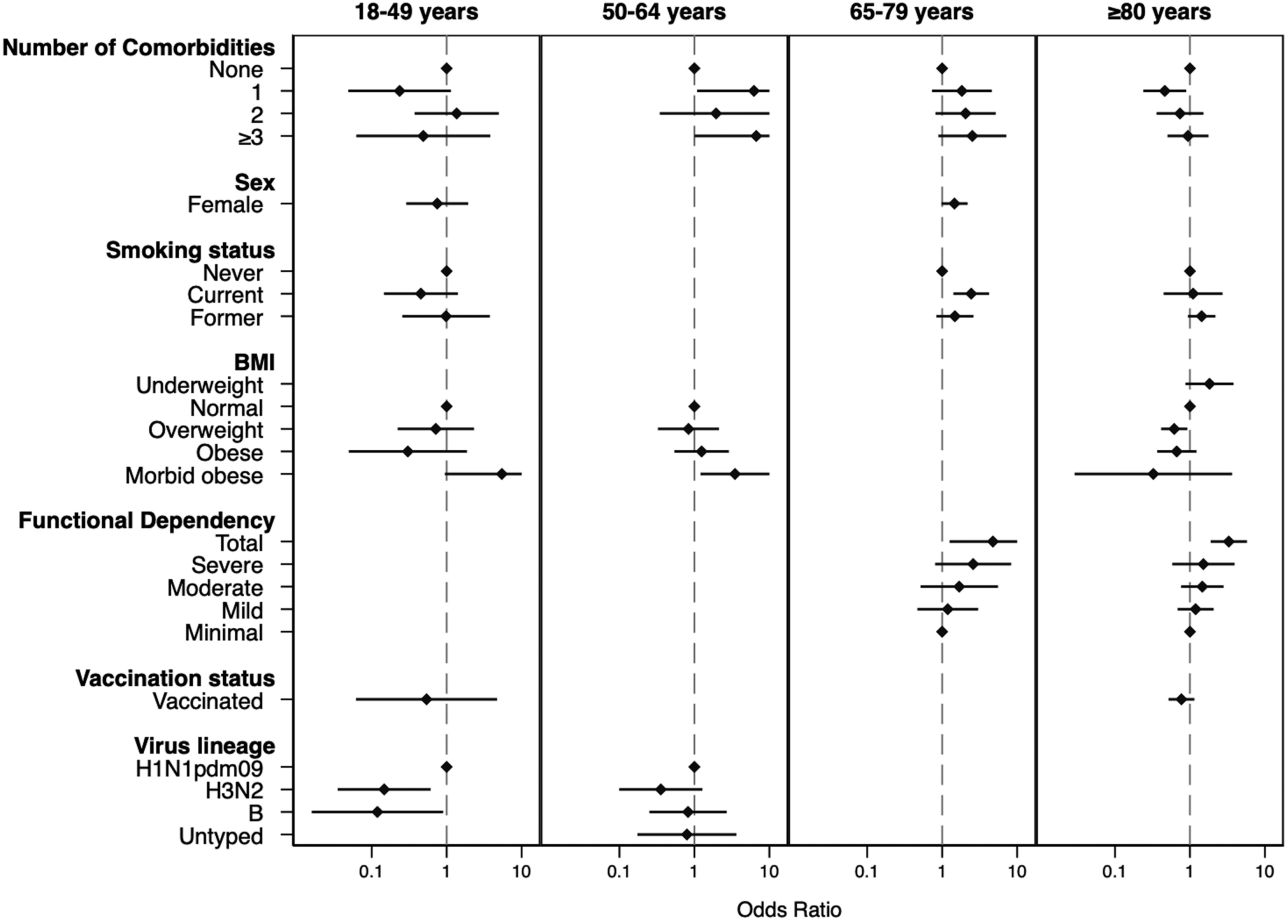
Multivariate risk factor analysis results from the four final GEE models. Odds ratios for severe influenza are represented on the *x*‐axis (Note: Some confidence intervals exceed the *x*‐axis scale). Study exposures are listed in the *y*‐axis

Presence of ≥3 comorbidities was associated with increased odds of severe influenza in the age groups 50–64 and 65–79, although the association was not consistently significant across stratum. Patients aged 50–64 with ≥3 comorbidities were almost seven times as likely to experience severe disease than those without comorbidity (OR = 6.7; 95% CI: 1.0–44.6; *P* = 0.05), and the odds of severe disease in those aged 65–79 were two and a half times higher (OR = 2.53; 95% CI: 0.89–7.16; *P* = 0.08) than their counterparts (Table 4 and Figure 1). Conversely, comorbidity did not appear to impact the odds of severity among those in the 18–49 and ≥80 age groups.

**TABLE 4:** Multivariate logistic regression analysis of risk factors associated with severe influenza among hospitalized adults with laboratory‐confirmed influenza, Valencia, 2010–2019

	Logistic regression parsimonious models											
	18–49 years			50–64 years			65–79 years			≥80 years		
	Odds ratio	95% confidence interval	*P*‐value	Odds ratio	95% confidence interval	*P*‐value	Odd ratio	95% confidence interval	*P*‐value	Odds ratio	95% confidence interval	*P*‐value
Number of comorbidities												
None	1.00	(Base)		1.00	(Base)		1.00	(Base)		1.00	(Base)	
1	0.24	0.05–1.14	0.072	6.23	1.09–35.60	0.040	1.83	0.73–4.59	0.195	0.46	0.24–0.89	0.021
2	1.36	0.37–4.96	0.640	1.95	0.35–10.97	0.450	2.05	0.82–5.17	0.127	0.74	0.36–1.51	0.405
≥3	0.49	0.06–3.82	0.495	6.70	1.01–44.60	0.049	2.53	0.89–7.16	0.081	0.94	0.50–1.76	0.851
Sex												
Female	0.75	0.29–1.94	0.551				1.46	0.98–2.18	0.063			
Smoking status												
Never	1.00	(Base)					1.00	(Base)		1.00	(Base)	
Current	0.45	0.15–1.41	0.173				2.44	1.41–4.22	0.001	1.10	0.45–2.72	0.833
Former	0.98	0.26–3.76	0.978				1.48	0.84–2.61	0.175	1.43	0.94–2.18	0.095
BMI												
Underweight	1.00	(Omitted)		1.00	(Omitted)					1.82	0.87–3.81	0.110
Normal	1.00	(Base)		1.00	(Base)					1.00	(Base)	
Overweight	0.72	0.22–2.31	0.578	0.83	0.33–2.13	0.704				0.62	0.41–0.92	0.018
Obese	0.30	0.05–1.86	0.198	1.25	0.54–2.88	0.601				0.67	0.37–1.22	0.190
Morbid obese	5.45	0.95–31.31	0.057	3.48	1.21–10.04	0.021				0.32	0.03–3.65	0.362
Functional dependency^a^												
Total							4.76	1.26–17.98	0.022	3.30	1.89–5.76	<0.0005
Severe							2.59	0.81–8.32	0.110	1.51	0.58–3.94	0.395
Moderate							1.69	0.51–5.55	0.387	1.46	0.76–2.81	0.252
Mild							1.19	0.47–3.01	0.717	1.19	0.69–2.07	0.536
Minimal							1.00	(Base)		1.00	(Base)	
Vaccination status												
Vaccinated	0.54	0.06–4.70	0.576							0.77	0.52–1.15	0.199
Virus lineage												
H1N1pdm09	1.00	(Base)			1.00	(Base)						
H3N2	0.15	0.04–0.61	0.008		0.36	0.10–1.28	0.113					
B	0.12	0.02–0.90	0.039		0.82	0.25–2.69	0.745					
Untyped	1.00	(Omitted)			0.79	0.17–3.63	0.766					

*Note*: Odds ratios not provided for those variables that dropped out final models, per each age group, according to model development specifications detailed above.

Abbreviation: BMI, body mass index.

^a^ Barthel Index data only available for patients ≥65 years. BMI categories were defined by underweight (<18.5), normal (18.5 to <25), overweight (25 to <30), obese (30 to <40) and morbid obese (≥40).

BMI had a varying effect across age groups. Morbidly obese patients showed increased odds of severe outcomes in the 50–64 age group, with an OR of 3.5 (1.2–10.0), whereas among patients ≥80 years, being overweight was associated with decreased odds of severe influenza (OR = 0.6; 0.4–0.9). Current smoking status increased the odds of severe outcome among patients aged 65–79 (OR = 2.4; 1.4–4.2). Functional status was an important risk factor for severity: Patients in the 65–79 age group with total dependency showed increased odds (OR = 4.8; 1.3–18.0) of severe influenza, similar to the ≥80 group (OR = 3.3; 1.9–5.8). In younger adults (18–49 years), infection with viral subtype/lineage A/H3N2 or B was associated with reduced severity when compared with H1N1pdm09 virus (OR = 0.2; 0.0–0.6; and OR = 0.1; 0.0–0.9, respectively).

### 3.4 Length of stay analysis

**FIGURE 2:**
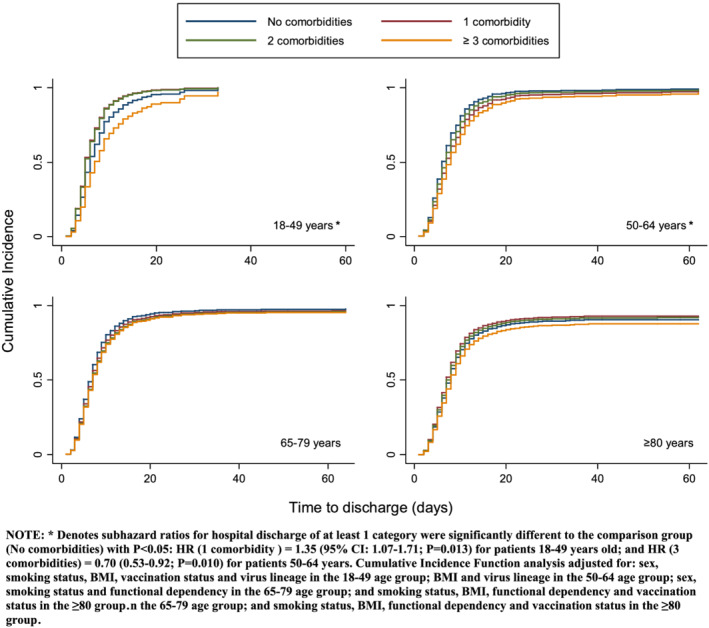
Time‐to‐discharge analysis with a cumulative incidence function using the method of Fine and Gray, per age strata and adjusted for confounders identified in each final GEE model, evaluating the effect of the comorbidities. Proportion of cumulated patients discharged at every time point represented on *y*‐axis. Time to discharge in days in *x*‐axis. *Subhazard ratios for hospital discharge of at least one category were significantly different to the comparison group (no comorbidities) with *P* < 0.05: HR (1 comorbidity) = 1.35 (95% CI: 1.07–1.71; *P* = 0.013) for patients 18–49 years old; and HR (3 comorbidities) = 0.70 (0.53–0.92; *P* = 0.010) for patients 50–64 years. Cumulative incidence function analysis adjusted for sex, smoking status, BMI, vaccination status and virus lineage in the 18–49 age group; BMI and virus lineage in the 50–64 age group; sex, smoking status and functional dependency in the 65–79 age group; and smoking status, BMI, functional dependency and vaccination status in the ≥80 group and the 65–79 age group; and smoking status, BMI, functional dependency and vaccination status in the ≥80 group

The median length of hospitalization was significantly longer for older than younger patients (test‐for‐trend across groups: *P* < 0.001; Table 2). Patients with ≥3 comorbidities were discharged later than those with fewer comorbidities irrespective of age, though these relationships appeared stronger in patients aged 18–49 and 50–64 years. The subdistribution hazard ratios for hospital discharge comparing patients with ≥3 versus no comorbidities were 0.73 (95% CI: 0.41–1.28; *P* = 0.271) for those aged 18–49 years, 0.70 (0.53–0.92; *P* = 0.010) for those 50–64 years old, 0.83 (0.68–1.02; *P* = 0.082) for those aged 65–79 years and 0.89 (0.72–1.11; *P* = 0.296) for patients ≥80 years old, after adjusting for potential confounders identified in GEE models and considering death a competing risk (Figure 2).

## 4 DISCUSSION

We identified associations between the prevalence of comorbidities and the frequency of severe outcomes among influenza hospitalized patients 50–79 years in Spain. BMI, virus strain, smoking and increased levels of functional dependency among the older population were also associated with influenza severity, albeit at different magnitudes across age groups. Patients with comorbidities and of older age also experienced longer hospitalization. Although respiratory outcomes were the most common discharge diagnoses across age groups, a considerable proportion of hospitalized patients with influenza had non‐respiratory outcomes as their primary discharge diagnosis, indicative of the range of clinical outcomes associated with influenza. Presence of comorbidity could not entirely explain disease severity in the young age group (18–49 years). Nonetheless, despite lack of risk factors, this group was vulnerable to severe disease outcomes due to H1N1pdm09 virus infection, with 20% having pneumonia as their main discharge diagnosis. This is a reminder that influenza virus infection can also lead to severe disease in otherwise healthy young adults who could benefit from influenza vaccination—which is not currently recommended for this age group in European countries.

We showed that severity increased steadily with age, from 6% among 18–49 years to 50% among those ≥80 years, with 26% of in‐hospital influenza death among those 65–79 years and 68% among ≥80 years (despite lower ICU admission rates in the latter age group, possibly a consequence of hospital care management decisions). Similarly, other studies have described that laboratory‐confirmed influenza cases have mortality rates increasing with ageing, although ICU admission would be requested more often for 40‐ to 79‐year age group than those ≥80 years.^27^ Older age, in its own right, is associated with deterioration of the immune system in producing an efficient response to infections or to developed immunity after vaccination, both of which are associated with mortality.^28^ However, the most challenging expression of population ageing is the clinical condition of frailty.^29^ It is estimated that as many as 50% of people ≥85 years are frail,^30^ which strongly predicts not only mortality but also cognitive decline, disability and institutionalization.^31^ Indeed, in our findings, in which we used functional dependency as a proxy for frailty, the odds of severe outcomes increased over threefold in the most frail individuals aged 65–79 and ≥80 years compared with those who were functionally independent at admission. Annual influenza vaccination can provide protection from severe influenza‐associated outcomes among older adults, including hospitalizations, ICU admissions and death even if the vaccine does not protect from infection.^32^ The availability of more immunogenic vaccines could have an even greater impact.

Comorbidities as predictors of influenza severity have been the subject of previous research, with reported higher risk of ICU admission and death in patients with specific comorbidities in the United States, mitigated by antiviral treatment or vaccination.^6^, ^32^ In our study, presence of comorbidity was associated with prolonged hospitalization, which drives influenza‐associated healthcare costs.^33^, ^34^ Another study of hospitalized influenza, using a similar severity definition, identified comorbidities such as diabetes and obesity to predispose complications in young adults aged 15–49 years; but these were less apparent in older adults.^7^ Our analysis suggests that the presence of comorbidities is an important risk factor among these and the 50‐ to 64‐year‐old patients, increasing their odds of severe clinical outcomes. Despite recommendation to vaccinate those with comorbidities, vaccination coverage in most countries are suboptimal.^9^ Weakening associations between comorbid status and severity in older adults are likely results of the high prevalence of comorbidities reducing their differentiating effect (87% of individuals aged ≥80 have ≥1 comorbidity) and may also reflect their increased risk stemming from other risk factors, as indicated by the association between severe outcome and functional dependency.

The effect of other exposures varied with age. Very high BMI was a risk factor for severity but being mildly overweight was protective in some categories. Indeed, the overall role of obesity remains unclear depending on categorization and settings, and it seems likely that underweight and heavily overweight status are more consistently associated with poor outcome.^7^, ^35^ Similarly, virus infection with H3N2 and B lineages showed to be less severe in age groups 18–49 and 50–64 compared with H1N1pdm09 strains, an effect that was larger in patients aged 18–49. Other studies have identified higher risk associated with H1N1pdm09 viruses in younger adults, probably a consequence of long‐term immunological memory to H1N1 viruses recalled in older adults infected in their youth.^36^, ^37^

In our study setting, only 16% of the patients was treated with antiviral medication, much lower than the United States, where over 80% of adults with influenza are treated.^38^ Unlike in the United States where influenza testing and treatment is more commonplace, in our study, testing was performed for research purposes, and tests results were not available to guide clinical management. Only 26% of all influenza positive cases were recorded as such in the discharge notes. The lack of recognition of influenza has public health implications and can lead to missed opportunities for treatment of high‐risk patients,^19^ especially when influenza vaccine uptake in high‐risk groups declines.^39^ In our study, though we included influenza vaccination status in our severity risk analysis, this did not survive model selection. Furthermore, we could not explore the effect of vaccination on severity of disease due to the potential for indication and health‐user biases that could not be addressed in the analysis.

Our study had some limitations. Despite the large dataset, severe outcomes are rare, which affected analytical power; moreover, deaths occurring after discharge were not captured. The final sample of severe laboratory‐confirmed influenza cases was therefore small, reducing the statistical power of the analysis and hindering our ability to assess further any potential impact of confounders. This was especially evident in the younger adult population, where case numbers were smallest. Our analysis was not designed to assess the impact of vaccination on severity, for which a different modelling strategy would have been required. Our modelling approach involves assumptions around data correlation, but results were qualitatively robust to sensitivity analyses using logistic or random effects models, increasing our confidence in reported results. Observational studies are affected by biases; for example, the decreased odds of severe outcomes in some group with one comorbidity may be a consequence of a lower clinical threshold for hospitalizing patients with comorbidities versus those with none, and interpretation of results needs to bear unmeasured confounders. Many individuals had more than one comorbidity, and we did not consider the impact of specific medical history on severity and could not assess whether some patients' comorbidities were better controlled than others', which could further affect interpretation.

In conclusion, our results confirm that influenza is an underappreciated disease that can cause severe clinical outcomes in adults of all ages, being further impacted by the presence of comorbidities and ageing. Increased awareness of influenza among hospitalized patients may have important impact for patients who could benefit from early antiviral therapy. Furthermore, availability of influenza vaccine formulations affording improved protection for adults, especially older adults and those with comorbidities, could minimize associated burden and healthcare resource consumption.

## AUTHOR CONTRIBUTIONS

**Nieves Derqui:** Formal analysis; visualization. **Joshua Nealon:** Conceptualization; formal analysis; methodology; supervision. **Ainara Mira‐Iglesias:** Conceptualization; data curation; methodology; validation. **JAVIER DIEZ‐DOMINGO:** Conceptualization; funding acquisition; methodology; supervision. **Cedric Mahe:** Funding acquisition; methodology; supervision. **Sandra S. Chaves:** Conceptualization; methodology; supervision.

### PEER REVIEW

The peer review history for this article is available at https://publons.com/publon/10.1111/irv.12985.

## Supporting information




**Table S1.** Discharge diagnoses groups created according to the major ICD categories, with the corresponding ICD‐9 and ICD‐10 codes assigned to each group. Description of the diseases is provided according to the ICD‐10 codes.
**Table S2.** Demographic and Clinical Characteristics of Hospitalized Adults with Laboratory‐Confirmed Influenza, Valencia, 2010–2019, age group distribution.

## Data Availability

The data that support the findings of this study are available from the authors upon reasonable request. The data are not publicly available due to privacy restrictions.
