# Supplementary material for: Predictors of influenza severity among hospitalized adults with laboratory confirmed influenza: Analysis of nine influenza seasons from the Valencia region, Spain
Source: Influenza Other Respir Viruses. 2022 Apr 12;16(5):862–72. doi: 10.1111/irv.12985 (PMC9343335; doi:10.1111/irv.12985)
Supplement: Supplementary file 1 — Table S1. Discharge diagnoses groups created according to the major ICD categories, with the corresponding ICD‐9 and ICD‐10 codes assigned to each group. Description of the diseases is provided according to the ICD‐10 codes. Table S2. Demographic and Clinical Characteristics of Hospitalized Adults with Laboratory‐Confirmed Influenza, Valencia, 2010–2019, age group distribution. [file IRV-16-862-s001.docx]

# Supplementary Tables

## Table S1

Table S1. Discharge diagnoses groups created according to the major ICD categories, with the corresponding ICD-9 and ICD-10 codes assigned to each group. Description of the diseases is provided according to the ICD-10 codes.

| Discharge diagnose | ICD-9 | ICD-10 | Description applied (according to ICD-10) |  |
| --- | --- | --- | --- | --- |
| Acute Upper Respiratory Infections | 460-466 | J00-J06 | Acute upper respiratory infections |  |
| Influenza | 487-488 | J09-J11 | Influenza |  |
| Pneumonia | 480-486 | J12-J18 | Pneumonia |  |
| Other Upper & Lower Respiratory Tract Diseases | 470-478 | J30-J39 | Other diseases of upper respiratory tract |  |
|  |  | J20-J22 | Other acute lower respiratory infections |  |
| Chronic Respiratory Diseases | 490-496 | J40-J47 | Chronic lower respiratory diseases |  |
| Other Respiratory Diseases | 500-508 | J60-J70 | Lung diseases due to external agents |  |
|  |  | J80-J84 | Other respiratory diseases principally affecting the interstitium |  |
|  |  | J85-J86 | Suppurative and necrotic conditions of the lower respiratory tract |  |
|  |  | J90-J94 | Other diseases of the pleura |  |
|  |  | J95 | Intraoperative and postprocedural complications and disorders of respiratory system, not elsewhere classified |  |
|  | 510-519 | J96-J99 | Other diseases of the respiratory system |  |
| Infectious Diseases | 001-139 | A00-B99 | Certain infectious and parasitic diseases | |
| Neoplasms | 140-239 | C00-D49 | Neoplasms | |
| Endocrine System Diseases | 240-279 | E00-E89 | Endocrine, nutritional and metabolic diseases | |
| Circulatory System Diseases | 280-289 | D50-D89 | Diseases of the blood and blood-forming organs and certain disorders involving the immune mechanism | |
|  | 390-459 | I00-I99 | Diseases of the circulatory system | |
| Mental Disorders | 290-319 | F01-F99 | Mental, Behavioural and Neurodevelopmental disorders | |
| Nervous System Diseases | 320-389 | G00-G99 | Diseases of the nervous system | |
|  |  | H00-H59 | Diseases of the eye and adnexa | |
|  |  | H60-H95 | Diseases of the ear and mastoid process | |
| Digestive System Diseases | 520-579 | K00-K95 | Diseases of the digestive system | |
| Genitourinary System Diseases | 580-629 | N00-N99 | Diseases of the genitourinary system | |
| Skin Tissue Diseases | 680-709 | L00-L99 | Diseases of the skin and subcutaneous tissue | |
| Musculoskeletal & Connective Tissue Diseases | 710-739 | M00-M99 | Diseases of the musculoskeletal system and connective tissue | |
| Pregnancy, Congenital Malformations & Other Related | 630-679 | O00-O9A | Pregnancy, childbirth and the puerperium | |
|  | 740-759 | Q00-Q99 | Congenital malformations, deformations and chromosomal abnormalities | |
|  | 760-779 | P00-P96 | Certain conditions originating in the perinatal period | |
| Various | 780-799 | R00-R99 | Symptoms, signs and abnormal clinical and laboratory findings, not elsewhere classified | |
|  | 800-999 | S00-T88 | Injury, poisoning and certain other consequences of external causes | |
|  | V01-V91 | V00-Y99 | External causes of morbidity | |
|  | E000-E999 | Z00-Z99 | Factors influencing health status and contact with health services | |

Table S2. Demographic and Clinical Characteristics of Hospitalized Adults with Laboratory-Confirmed Influenza, Valencia, 2010–2019, age group distribution.

|  |  | 18-49 years patients, No. (%) | | | 50-64 years patients, No. (%) | | | 65-79 years patients, No. (%) | | | ≥80 years patients, No. (%) | | |
| --- | --- | --- | --- | --- | --- | --- | --- | --- | --- | --- | --- | --- | --- |
| Characteristics | | **Not Severe (n=287)** | **Severe (n=16)** | ***P* value** | **Not Severe (n=451)** | **Severe (n=37)** | ***P* value** | **Not Severe (n=1107)** | **Severe (n=84)** | ***P* value** | **Not Severe (n=1065)** | **Severe (n=135)** | ***P* value** |
| Number of Comorbidities | |  |  | 0.190 |  |  | 0.015 |  |  | 0.097 |  |  | 0.053 |
|  | None | 118 (41) | 10 (63) |  | 100 (22) | 2 (6) |  | 136 (12) | 4 (5) |  | 106 (10) | 17 (13) |  |
|  | 1 | 113 (39) | 2 (13) |  | 158 (35) | 17 (49) |  | 320 (29) | 22 (26) |  | 304 (29) | 25 (19) |  |
|  | 2 | 43 (15) | 3 (19) |  | 112 (25) | 5 (14) |  | 288 (26) | 22 (26) |  | 316 (30) | 39 (29) |  |
|  | ≥3 | 13 (5) | 1 (6) |  | 81 (18) | 11 (31) |  | 363 (33) | 36 (43) |  | 339 (32) | 54 (40) |  |
| Sex | |  |  | 0.903 |  |  | 0.943 |  |  | 0.313 |  |  | 0.923 |
|  | Male | 139 (48) | 8 (50) |  | 242 (54) | 19 (54) |  | 681 (62) | 47 (56) |  | 478 (45) | 60 (44) |  |
|  | Female | 148 (52) | 8 (50) |  | 209 (46) | 16 (46) |  | 426 (38) | 37 (44) |  | 587 (55) | 75 (56) |  |
| Smoking | |  |  | 0.903 |  |  | 0.630 |  |  | 0.286 |  |  | 0.762 |
|  | Current | 113 (39) | 6 (37) |  | 174 (39) | 11 (31) |  | 168 (15) | 18 (21) |  | 45 (4) | 6 (4) |  |
|  | Former | 42 (15) | 3 (19) |  | 163 (36) | 13 (37) |  | 471 (43) | 35 (42) |  | 308 (29) | 43 (32) |  |
|  | Never | 132 (46) | 7 (44) |  | 114 (25) | 11 (31) |  | 468 (42) | 31 (37) |  | 712 (67) | 86 (64) |  |
| BMI | |  |  | 0.044 |  |  | 0.091 |  |  | 0.962 |  |  | 0.040 |
|  | Underweight | 12 (4) | 0 (0) |  | 5 (1) | 0 (0) |  | 20 (2) | 2 (2) |  | 19 (2) | 6 (4) |  |
|  | Normal | 113 (39) | 6 (37) |  | 126 (28) | 8 (23) |  | 293 (26) | 24 (29) |  | 321 (30) | 53 (39) |  |
|  | Overweight | 83 (29) | 5 (31) |  | 177 (39) | 10 (29) |  | 460 (42) | 32 (38) |  | 442 (42) | 45 (33) |  |
|  | Obese | 69 (24) | 2 (12) |  | 116 (26) | 11 (31) |  | 302 (27) | 24 (29) |  | 268 (25) | 30 (22) |  |
|  | Morbid obese | 10 (3) | 3 (19) |  | 27 (6) | 6 (17) |  | 32 (3) | 2 (2) |  | 15 (1) | 1 (1) |  |
| Functional Dependency * | |  |  | NA |  |  | NA |  |  | 0.002 |  |  | *P<0.001* |
|  | Total | NA | NA |  | NA | NA |  | 26 (2) | 8 (10) |  | 92 (9) | 28 (21) |  |
|  | Severe | NA | NA |  | NA | NA |  | 15 (1) | 2 (2) |  | 44 (4) | 6 (4) |  |
|  | Moderate | NA | NA |  | NA | NA |  | 28 (3) | 3 (4) |  | 100 (9) | 13 (10) |  |
|  | Mild | NA | NA |  | NA | NA |  | 131 (12) | 12 (14) |  | 295 (28) | 35 (26) |  |
|  | Minimal | NA | NA |  | NA | NA |  | 907 (82) | 59 (70) |  | 534 (50) | 53 (39) |  |
| Cardiovascular Disease | |  |  | 0.291 |  |  | 0.065 |  |  | 0.290 |  |  | 0.968 |
|  | Yes | 17 (6) | 2 (12) |  | 127 (28) | 15 (43) |  | 540 (49) | 46 (55) |  | 645 (61) | 82 (61) |  |
|  | No | 270 (94) | 14 (88) |  | 324 (72) | 20 (57) |  | 567 (51) | 38 (45) |  | 420 (39) | 53 (39) |  |
| Asthma | |  |  | 0.417 |  |  | 0.081 |  |  | 0.599 |  |  | 0.872 |
|  | Yes | 60 (21) | 2 (12) |  | 58 (13) | 1 (3) |  | 112 (10) | 7 (8) |  | 75 (7) | 9 (7) |  |
|  | No | 227 (79) | 14 (88) |  | 393 (87) | 34 (97) |  | 995 (90) | 77 (92) |  | 990 (93) | 126 (93) |  |
| Other Chronic Respiratory Disease | |  |  | 0.382 |  |  | 0.807 |  |  | 0.158 |  |  | 0.273 |
|  | Yes | 40 (14) | 1 (6) |  | 171 (38) | 14 (40) |  | 415 (37) | 38 (45) |  | 314 (29) | 46 (34) |  |
|  | No | 247 (86) | 15 (94) |  | 280 (62) | 21 (60) |  | 692 (63) | 46 (55) |  | 751 (71) | 89 (66) |  |
| Chronic Endocrine System Disease | |  |  | 0.996 |  |  | 0.011 |  |  | 0.652 |  |  | 0.956 |
|  | Yes | 36 (13) | 2 (12) |  | 127 (28) | 17 (49) |  | 499 (45) | 40 (48) |  | 384 (36) | 49 (36) |  |
|  | No | 251 (87) | 14 (88) |  | 324 (72) | 18 (51) |  | 608 (55) | 44 (52) |  | 681 (64) | 86 (64) |  |
| Anaemia | |  |  | 0.329 |  |  | 0.867 |  |  | 0.648 |  |  | 0.451 |
|  | Yes | 18 (6) | 2 (12) |  | 29 (6) | 2 (6) |  | 95 (9) | 6 (7) |  | 148 (14) | 22 (16) |  |
|  | No | 269 (94) | 14 (88) |  | 422 (94) | 33 (94) |  | 1012 (91) | 78 (93) |  | 917 (86) | 113 (84) |  |
| Chronic Liver Disease | |  |  | 0.384 |  |  | 0.136 |  |  | 0.278 |  |  | 0.054 |
|  | Yes | 13 (5) | 0 (0) |  | 27 (6) | 0 (0) |  | 40 (4) | 5 (6) |  | 20 (2) | 6 (4) |  |
|  | No | 274 (95) | 16 (100) |  | 424 (94) | 35 (100) |  | 1067 (96) | 79 (94) |  | 1045 (98) | 129 (96) |  |
| Chronic Renal Disease | |  |  | 0.629 |  |  | 0.065 |  |  | 0.006 |  |  | 0.162 |
|  | Yes | 11 (4) | 1 (6) |  | 45 (10) | 7 (20) |  | 164 (15) | 22 (26) |  | 206 (19) | 33 (24) |  |
|  | No | 276 (96) | 15 (94) |  | 406 (90) | 28 (80) |  | 943 (85) | 62 (74) |  | 859 (81) | 102 (76) |  |
| Immunopathology | |  |  | 0.228 |  |  | 0.664 |  |  | 0.026 |  |  | 0.845 |
|  | Yes | 24 (8) | 0 (0) |  | 30 (7) | 3 (9) |  | 47 (4) | 8 (10) |  | 36 (3) | 5 (4) |  |
|  | No | 263 (92) | 16 (100) |  | 421 (93) | 32 (91) |  | 1060 (96) | 76 (90) |  | 1029 (97) | 130 (96) |  |
| Neurological Disorders | |  |  | 0.448 |  |  | 0.373 |  |  | 0.630 |  |  | 0.341 |
|  | Yes | 10 (3) | 0 (0) |  | 10 (2) | 0 (0) |  | 67 (6) | 4 (5) |  | 149 (14) | 23 (17) |  |
|  | No | 277 (97) | 16 (100) |  | 441 (98) | 35 (100) |  | 1040 (94) | 80 (95) |  | 916 (86) | 112 (83) |  |
| Neoplasia | |  |  | 0.442 |  |  | 0.601 |  |  | 0.596 |  |  | 0.088 |
|  | Yes | 10 (4) | 0 (0) |  | 27 (8) | 3 (10) |  | 78 (9) | 5 (7) |  | 38 (4) | 9 (8) |  |
|  | No | 219 (96) | 13 (100) |  | 327 (92) | 26 (90) |  | 787 (91) | 65 (93) |  | 814 (96) | 101 (92) |  |
| Vaccination Status | |  |  | 0.280 |  |  | 0.518 |  |  | 0.629 |  |  | 0.320 |
|  | Yes | 47 (16) | 1 (6) |  | 153 (34) | 10 (29) |  | 709 (64) | 56 (67) |  | 739 (69) | 88 (65) |  |
|  | No | 240 (84) | 15 (94) |  | 298 (66) | 25 (71) |  | 398 (36) | 28 (33) |  | 326 (31) | 47 (35) |  |
| Antiviral Use | |  |  | 0.290 |  |  | 0.955 |  |  | 0.919 |  |  | 0.552 |
|  | Yes | 58 (20) | 5 (31) |  | 79 (18) | 6 (17) |  | 176 (16) | 13 (15) |  | 146 (14) | 16 (12) |  |
|  | No | 229 (80) | 11 (69) |  | 372 (82) | 29 (83) |  | 931 (84) | 71 (85) |  | 919 (86) | 119 (88) |  |
| Virus strain | |  |  | 0.010 |  |  | 0.140 |  |  | 0.578 |  |  | 0.890 |
|  | H1N1pdm09 | 112 (39) | 13 (81) |  | 193 (43) | 21 (60) |  | 237 (21) | 23 (27) |  | 130 (12) | 18 (13) |  |
|  | H3N2 | 114 (40) | 2 (12) |  | 193 (43) | 8 (23) |  | 652 (59) | 45 (54) |  | 733 (69) | 93 (69) |  |
|  | B | 47 (16) | 1 (6) |  | 43 (10) | 4 (11) |  | 176 (16) | 12 (14) |  | 163 (15) | 18 (13) |  |
|  | Untyped | 14 (5) | 0 (0) |  | 22 (5) | 2 (6) |  | 42 (4) | 4 (5) |  | 39 (4) | 6 (4) |  |
| Season | |  |  | 0.234 |  |  | 0.105 |  |  | *P<0.001* |  |  | 0.076 |
|  | 2010/11 | 21 (7) | 3 (19) |  | 29 (6) | 3 (9) |  | 32 (3) | 1 (1) |  | 8 (1) | 1 (1) |  |
|  | 2011/12 | 37 (13) | 0 (0) |  | 68 (15) | 3 (9) |  | 210 (19) | 13 (15) |  | 205 (19) | 24 (18) |  |
|  | 2012/13 | 29 (10) | 0 (0) |  | 23 (5) | 1 (3) |  | 75 (7) | 3 (4) |  | 53 (5) | 3 (2) |  |
|  | 2013/14 | 54 (19) | 6 (37) |  | 80 (18) | 6 (17) |  | 89 (8) | 7 (8) |  | 57 (5) | 4 (3) |  |
|  | 2014/15 | 34 (12) | 1 (6) |  | 68 (15) | 1 (3) |  | 243 (22) | 10 (12) |  | 275 (26) | 26 (19) |  |
|  | 2015/16 | 33 (11) | 2 (12) |  | 50 (11) | 4 (11) |  | 86 (8) | 2 (2) |  | 58 (5) | 10 (7) |  |
|  | 2016/17 | 17 (6) | 0 (0) |  | 20 (4) | 0 (0) |  | 74 (7) | 5 (6) |  | 100 (9) | 15 (11) |  |
|  | 2017/18 | 41 (14) | 3 (19) |  | 68 (15) | 10 (29) |  | 196 (18) | 24 (29) |  | 192 (18) | 39 (29) |  |
|  | 2018/19 | 21 (7) | 1 (6) |  | 45 (10) | 7 (20) |  | 102 (9) | 19 (23) |  | 117 (11) | 13 (10) |  |

**NOTE: * Barthel Index data only available for patients ≥65 year. BMI categories were defined by underweight (<18.5), normal (18.5 to <25), overweight (25 to <30), obese (30 to <40), and morbid**

**obese (≥40). Abbreviations: BMI, Body Mass Index.**
